# Success and Complication Rates of Revascularization Procedures for Immature Necrotic Teeth: A Systematic Review

**DOI:** 10.7759/cureus.51364

**Published:** 2023-12-30

**Authors:** Lalitha Priya B, Neha Singh, Keshav Kumar Mangalam, Rohan Sachdev, Aishwarrya P, Harshvardhan N Jain, Puneet Kamal Nagi

**Affiliations:** 1 Conservative Dentistry and Endodontics, Sri Ramakrishna Dental College and Hospital, Coimbatore, IND; 2 Conservative Dentistry and Endodontics, Maharana Pratap Dental College and Hospital, Kanpur, IND; 3 Public Health, The University of Western Australia Dental School, Crawley, AUS; 4 Orthodontics and Dentofacial Orthopaedics, Sri Ramakrishna Dental College and Hospital, Coimbatore, IND; 5 Dentistry, Terna Dental College and Hospital, Navi Mumbai, IND; 6 Periodontics, Punjab Government Dental College and Hospital, Amritsar, IND

**Keywords:** regenerative endodontic treatment, platelet-rich fibrin (prf), platelet-rich plasma (prp), immature tooth, immature necrotic teeth, apexification

## Abstract

Frequently, adolescents exhibit instances of immature necrotic teeth, which are identifiable by their slender root walls and unclosed root tips. The lack of a natural narrowing near the root's end creates difficulty when using standard endodontic procedures, making the effective sealing of the immature root canal difficult or impractical. Revascularization therapy surfaces as a prospective strategy for addressing the management of undeveloped, non-vital, immature, necrotic teeth. Notwithstanding this, apexification continues to hold prominence in the preferences of clinicians owing to its perceived predictability in treatment outcomes.

A systematic investigation was conducted involving various search engines and databases, covering the period from 2001 to 2023. The main aim of this investigation was to find randomized clinical trials that compared the efficacy of revascularization therapy to apexification for treating immature necrotic teeth. The evaluation included a thorough examination of both clinical and radiographic outcomes assessing the success rates and complications. Out of the 850 identified articles, 15 studies were chosen for comprehensive analysis. Notable dissimilarities were not identified between the revascularization therapy and apexification groups concerning parameters such as rates of periapical healing, overall effectiveness/invalidation, and apical closure. However, concentrating on measurable factors, it became clear that the revascularization treatment group displayed a notable rise in root length compared to the apexification group.

Both revascularization endodontic therapy and apexification demonstrated effectiveness in addressing periapical periodontitis healing and open apex closure. Pulp revascularization stood out for its notable efficacy in enhancing root elongation and thickening, all while having a reduced likelihood of treatment being deemed ineffective overall.

## Introduction and background

Addressing the ongoing issue of managing immature permanent teeth that have incomplete root formation has consistently posed a challenge in the realm of endodontic treatment. These early permanent teeth have a spacious pulp chamber and an open apex, making them susceptible to fractures due to the delicate and slender dentinal walls in their root canals [1]. The process of maturation for these immature teeth spans approximately three to five years until their roots achieve full development. However, during this period, instances of pulp necrosis or periapical inflammation present significant difficulties [2].

Regenerative endodontics is created on three fundamental principles rooted in tissue engineering. These principles involve identifying suitable sources of stem/progenitor cells, delivering growth factors to direct the differentiation of these cells, and establishing a supportive three-dimensional structure that promotes continuous cellular growth and specialization [3]. The main reservoirs of stem cells are located in the apical papilla [4] and the periapical tissues of developing permanent teeth [5,6].

Stem cells are categorized into embryonic stem cells and adult stem cells, commonly known as postnatal cells [6]. In the context of dental pulp regeneration, mature stem cells play a critical role. These cells are distributed throughout the dental structure, including the pulp, apical papilla, as well as periodontal ligament [7]. Possessing a propensity for rapid differentiation, these cells have the capacity to orchestrate the renewal of dentin and pulp tissue under the influence of appropriate differentiation signals [6,7].

Because dental pulp originates from the neural crest, the regeneration of nerves is also supported. It is crucial for maintaining the vitality of stem cells and directing their paths of differentiation. This is where growth factors come into play, frequently released by platelets and other cellular constituents found within blood clots [8]. The necessary structural support is naturally furnished by intracanal blood clots, dentin walls, and the fibrin network formed through platelet-induced coagulation.

Pulp revitalization hinges on the inherent capability of remaining pulp and periodontal stem cells to undergo differentiation [9,10]. This process contributes to the development of a living matrix rich in vascularized and connective tissue. This matrix occupies the available pulp space and steers stem cell differentiation into newly formed odontoblasts, initiating the deposition of hard tissue with characteristics yet to be fully determined [11]. Revascularization introduces an innovative approach to tackle the issue of immature necrotic permanent teeth, aiming to restore vitality and support complete root development. Traditionally, apexification has been the established method for such cases. This method involves inducing calcification to create a barrier at the exposed apex, often utilizing calcium hydroxide. This procedure establishes an environment conducive to periapical tissue regeneration and facilitates subsequent root canal treatment [12].

In contrast, revascularization promotes continued root growth, contrasting with conventional apexification methods. It not only reduces the dimensions of the immature tooth's apical opening but also enhances apical development and comprehensive root maturation, encompassing root elongation, dentinal wall thickening, and natural apex formation [11,12].

Revascularization is recommended in cases where deep caries or trauma has disrupted the normal root canal development in immature teeth. The typical alternative involves performing an endodontic procedure on an immature tooth with an open apex, resulting in structural compromise [5].

Pulp revascularization is a multi-step process that includes a comprehensive cleaning of the root canal, the stimulation of bleeding to supply essential growth factors and stem cells, the placement of a compatible scaffold, sealing the tooth, and regular monitoring. The scaffold can consist of materials like a blood clot, platelet-rich plasma (PRP), or other suitable substances, creating a supportive framework for tissue regeneration. Over time, the tooth naturally matures, with the root elongating, the dentinal walls thickening, and the apex forming. Pulp revascularization offers a promising alternative to conventional apexification methods, maintaining the tooth's function and structure while fostering continuous growth and vitality. Despite overall pulp vitality loss, residual stem cells within the pulp and apical papilla endure, facilitated by abundant blood supply within the apical region [5,13].

Two distinct methodologies for pulp revascularization have been documented. One approach utilizes calcium hydroxide, while the other employs a triple antibiotic paste [11]. Both methods follow a two-step procedure designed to disinfect the necrotic pulp. Approximately two to three weeks after the initial step, the second phase is initiated, contingent on the absence of symptoms and visible reduction in apical lesion size [13]. Around three months post-procedure, the absence of symptoms is typically observed, and the radiographic assessment nine months later shows increased dentinal wall thickness and apical closure. Observable root development and apical closure may manifest as early as three months post-treatment [5].

Effective sealing, a supporting matrix, and root canal disinfection are necessary for successful revascularization. PRP provides concentrated growth factors, which help in regeneration, whereas blood clots function as scaffolds but lack growth factors. Unlike PRP, which needs to be processed biochemically, platelet-rich fibrin (PRF) is solely autologous and easier to manufacture, making it an excellent scaffold. PRF is a potential scaffolding material for regenerative endodontic operations because of its simplicity and autologous origin [5,6].

Effectively managing periapical inflammation is a crucial goal when dealing with immature necrotic teeth. As a result, conducting a systematic review to assess the effectiveness of treatments designed to promote periapical healing becomes essential.

## Review

Materials and methodology

This systematic review adhered to the recommendations outlined in the Preferred Reporting Items for Systematic Reviews and Meta-Analyses (PRISMA) guidelines. Prior to initiating the review process, a methodology was devised in accordance with the guidance provided by the Cochrane Handbook for Systematic Reviews of Interventions.

Focused Questions

Is revascularization more successful than apexification at treating immature necrotic teeth's periapical healing outcomes? Does revascularization produce better results for root development when treating young necrotic teeth than apexification does?

Search Strategy

We adopted an interdisciplinary approach in our methodological design, which encompassed a blend of cohort studies, case-control studies, and randomized clinical trials. Our scrutiny of relevant data for this review included a meticulous examination of primary research articles, review articles, published bibliographies, and pertinent citations. This endeavour was specifically directed at acquiring a collection of randomized clinical trials.

To search for relevant articles, a comprehensive search was carried out on databases including MEDLINE/PubMed, Cochrane, and EMBASE (Excerpta Medica Database). Articles published from 2001 to 2023 were gathered, with no limitations placed on language or publication year. Throughout this search process, MeSH terms such as "immature teeth", "young permanent teeth", "revascularization", "regenerative endodontics", "apexification", "root end formation", and "apical closure" were employed as the search keywords.

**Figure 1:**
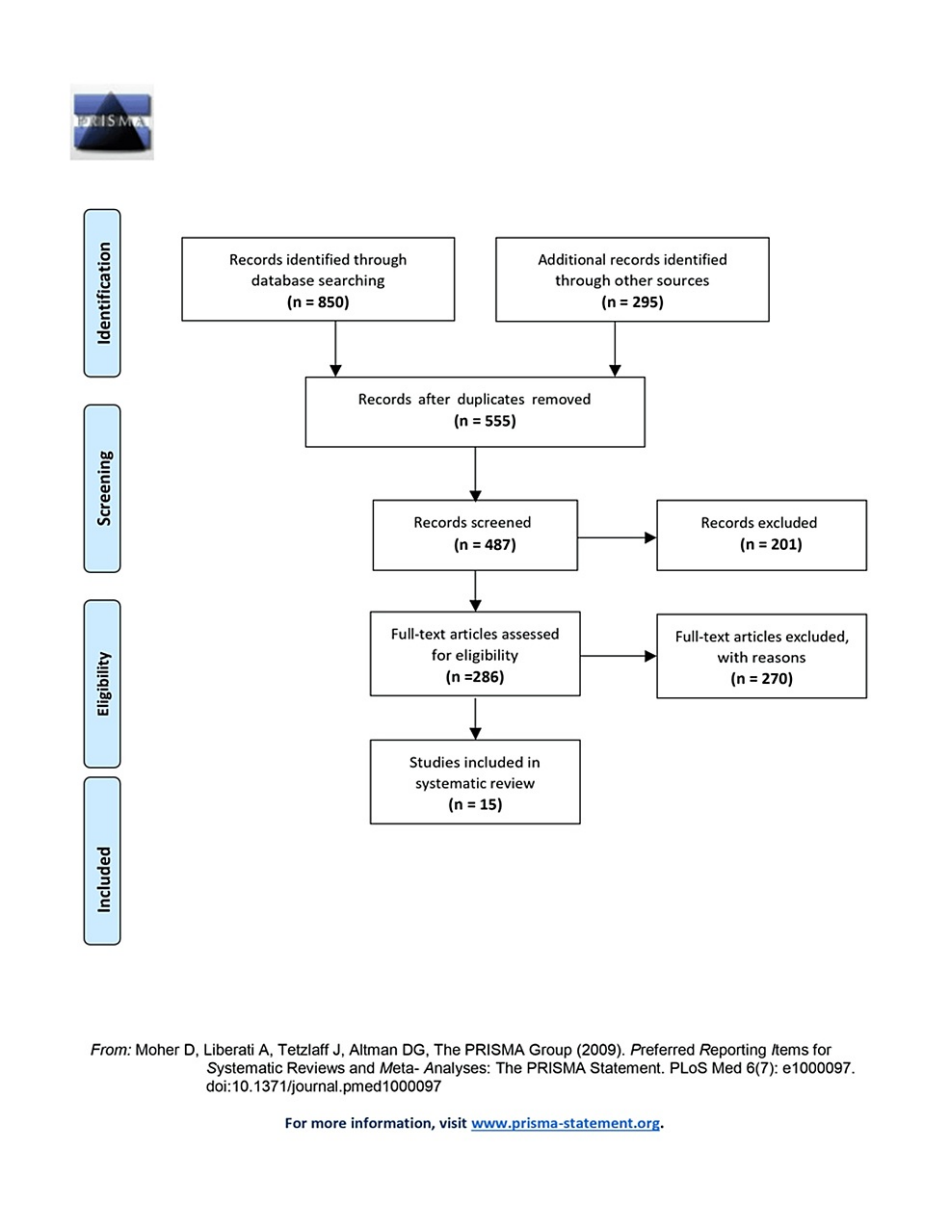
PRISMA flowchart detailing the study identification and selection process. PRISMA: Preferred Reporting Items for Systematic Reviews and Meta-Analyses

The criteria for study selection and exclusion were defined in accordance with the PRISMA checklist as shown in Figure 1. The researchers meticulously evaluated the complete texts of the studies and independently assessed them against the pre-established inclusion criteria. The strict adherence to the PRISMA statement guidelines, combined with the execution of the pre-defined search strategy as below, was thoroughly maintained. Furthermore, a manual search of the reference sections of the incorporated studies was conducted to ensure a comprehensive coverage of the available literature.

The established criteria for inclusion in this analysis were as follows: The research encompassed controlled clinical trials (CCTs) and randomized controlled trials (RCTs) and it encompassed works composed in the English language.

Conversely, the following were excluded from the investigation: animal studies, in vitro research, case studies, and research with a retrospective design conducted in real time. The determination of inclusion and exclusion criteria was guided by the aspects of Study design, Participants, Interventions, Comparisons, and Outcomes (SPICO) as shown in Table 1.

**Table 1:** PICO criteria: The PICO framework focuses on the Population, Intervention, Comparison, and Outcomes, and it is a commonly used tool for quantitative systematic review.

Population	Individuals dealing with necrotic immature permanent teeth.
Intervention	Treatment involving regenerative endodontics
Comparison	Treatment centred around apexification
Outcome	Documented results from clinical and radiographic assessments
Study design	Randomized clinical trials

Screening and Selection

The process of search and screening was executed collaboratively by two researchers, and the degree of concordance between them was quantified through a κ coefficient of 0.83, indicating a substantial level of agreement. The articles identified through the search underwent a comprehensive evaluation to determine their suitability for inclusion or exclusion, following a structured framework of four distinct stages.

During the initial Stage 1, citations lacking relevance were promptly excluded from further consideration. Progressing to Stage 2, a singular reviewer meticulously assessed the titles and abstracts of all retrieved articles to ascertain their alignment with the predefined criteria. Articles that manifestly fell outside the scope of inclusion parameters were promptly excluded, while those that raised ambiguity underwent a thorough scrutiny of their full content. In cases of uncertainty, a second reviewer's input was sought to inform the evaluation.

Advancing to Stage 3, all articles selected during Stage 1 underwent a meticulous assessment by two independent reviewers to validate their alignment with the established eligibility criteria. This phase encompassed the exclusion of articles possessing inappropriate study designs or exhibiting inadequacies in the measurement of outcomes at both baseline and endpoint. Articles demonstrating deficiencies in referencing were also excluded.

Finally, during Stage 4, all articles considered suitable for inclusion underwent a thorough examination, with pertinent data being extracted from each. The clinical methodologies employed in all scrutinized studies were critically appraised, centring on the specifics of interventions and outcomes explored within each individual study.

Data Extraction

The initial phase of data extraction was conducted by the principal author, followed by a review and refinement process overseen by the second author. Independent data extraction was executed for every full-text article that satisfied the predetermined inclusion criteria. This procedure adhered to a standardized format facilitated by digital tools (Microsoft Office Excel 2013 software, Microsoft Corporation, Redmond, Washington, United States), as elaborated upon in Table 2. The amassed data were methodically organized into separate sections, encompassing attributes like authorship and year of publication, study design, participant demographic details, age range, specifics of interventions, comparator elements, and resulting outcomes.

**Table 2:** Data extraction sheet PRP: Platelet-rich plasma; PRF: Platelet-rich fibrin; BLC: Blood clot control; PLGA: Poly‑lactic co‑glycolic acid

Study	Population	Type of Study	The age range of patients	Parameters checked	Intervention	Comparison	Outcome	Time period
Lin et al. (2017) [13]	103 patients	Prospective Randomized Controlled Study	6 -18 years	Clinical and radiographic examinations	Triple and double antibiotic paste as intracanal medicament, calcium hydroxide, absorbable collagen barrier	To measure the change of root length, root thickness, and apical foramen size at the 12-month follow-up	Calcium hydroxide increases root length in relation to blood clots, favours apex closure	12 months
Saravanan et al. (2017) [14]	25 healthy subjects	A Double-Blinded Randomized Control	Less than 18 years	Clinical and radiographic examinations	Triple and double antibiotic paste as intracanal medicament, mineral trioxide aggregate, blood clot	Assessed at baseline, 6 months, and 12 months	The increase in the root length is superior over the study period in the triple antibiotic arm when compared to that of the double antibiotic arm	12 months
Mohamed et al. (2014) [15]	36 patients with immature, nonvital maxillary anterior teeth	Randomized Controlled Trials	9–13 years	Clinical and radiographic examinations	Triple antibiotic paste as intracanal medicament, mineral trioxide aggregate, blood clot, fibroblast growth factor	Evaluation of root length, and apical closure periodically till 18 months	After a follow-up period of 18 months, most of the cases showed radiographic evidence of periapical healing.	18 months
Huang et al. (2013) [16]	36 endodontic infection teeth with immature tooth	Randomized Controlled Trials	7-13 years	Clinical and radiographic examinations	Metronidazole as intracanal medicament, calcium hydroxide, blood clot	Healing of the lesion was evaluated every month until 18 months	Blood clot increases root length in relation to calcium hydroxide, favours apex closure	18 months
Narang et al. (2015) [17]	20 Young healthy subjects below 20 years of age	Randomized Controlled Trials	7 – 20 years	Clinical and radiographic examinations	Triple antibiotic paste as an intracanal medicament, mineral trioxide aggregate, blood clot, plasma-rich protein, plasma-rich fibrin	Evaluation after 6 and 18 months was done by the groups.	PRF has huge potential to accelerate the growth characteristics in immature necrotic permanent teeth as compared to PRP and blood clots.	12 months
Jadhav et al., (2012) [18]	20 patients with nonvital, immature anterior teeth	Randomized Controlled Trials	15–28 years	Clinical and radiographic examinations	Triple antibiotic paste as intracanal medicament, blood clot and platelet concentration in PRP	The evaluation was done after 6 and 12 months	PRP supplement improved the outcome of revascularization	12 months
Alagl et al. (2017) [19]	30 non-vital immature permanent teeth	Randomized Controlled Trials, split-mouth	7–12 years	Clinical and radiographic examinations, CT	Triple antibiotic paste as intracanal medicament, platelet concentration in PRP	The evaluation was done after the 3^rd^ week followed by 5 months and then 12 months	PRP can serve as a successful scaffold for regenerative endodontic treatment	12 months
Sharma et al. (2016) [20]	16 immature permanent teeth	Randomized Controlled Trials	10–25 years	Clinical and radiographic examinations	Plasma-rich plasma, collagen, PLGA	Evaluation is done at 6 and 12 months compared with the baseline	PRF and collagen are better scaffolds than blood clots and PLGA for inducing apexogenesis in immature necrotic permanent teeth	12 months
Shivashankar et al. (2017) [21]	60 patients with necrotic immature permanent tooth	Triple Blind Randomized Clinical Trial	6 to 28 years	Clinical: pulpal sensitivity test and radiographic examinations	Triple antibiotic paste as an intracanal medicament, Group A-PRF, Group B-induced bleeding technique, Group C-PRP	Evaluation at baseline, 3rd, 6th, 9th, and at 12 months	Establish induced bleeding technique as the standard endodontic procedure for revascularization	12 months
Mittal et al. (2021) [22]	16 patients with necrotic immature permanent tooth	Randomized Clinical Trial, Parallel	9–14 years	Clinical and radiographic examinations	Double antibiotic paste as an intracanal medicament, PRF, collagen, Placentrex, and chitosan in four groups	Evaluation done at 3, 6, and 12 months and compared with baseline	PRF and collagen are better scaffolds than Placentrex and chitosan for inducing apexogenesis	12 months
Ragab et al. (2019) [23]	22 patients with immature necrotic permanent maxillary central incisors	Randomized Clinical Trial, Parallel, Double-blinded	7–12 years	Clinical and radiographic examinations	Double antibiotic paste as an intracanal medicament, blood clot and PRF	Evaluation done at 6 and 12 months and compared with baseline	The use of PRF was not essential for repair	12 months
Ulusoy et al. (2019) [24]	67 healthy children with 88 immature necrotic incisors	Prospective Randomized Trial	8-11 years	Clinical and radiographic examinations	Double antibiotic paste as an intracanal medicament, blood clot, PRP, PRF, and platelet pellet	Evaluate clinically every 3 months and radiographically every 6 months	PRP, PRF, and plasma pellet yield similar clinical and radiographic outcomes to blood clot	10–49 months
ElSheshtawy et al. (2020) [25]	26 healthy patients with immature permanent anterior teeth with necrotic pulps	Randomized Controlled Trial	12.66 ± 4.77 years	Clinical and radiographic examinations	Triple antibiotic paste as an intracanal medicament, PRP as a scaffold, and a test group	Evaluation done at 6 and 12 months and compared with baseline	Successful outcomes of regenerative endodontic technique using PRP and BLC as scaffolds in immature traumatized permanent teeth	12 months
Rizk et al. (2019) [26]	30 immature maxillary anterior permanent incisors	Split Mouth Double-blinded Randomized Controlled Trial	8–14 years	Clinical and radiographic examinations	Triple antibiotic paste as an intracanal medicament, PRP as a scaffold, and a test group	Evaluation was done at 3, 6, 9, and 12 months.	Revascularization using PRP is a desirable alternative to a blood clot	12 months
Ramachandran et al. (2020) [27]	40 teeth with open apices in patients	Randomized Clinical Trial	15-54 years	Clinical and radiographic examinations	Triple antibiotic paste as an intracanal medicament, groups using blood clot and plasma-rich plasma	Evaluation was done at 6 and 12 months.	Revascularization procedure provides several potential benefits over conventional root canal treatment	12 months

Assessment of Risk of Bias

In order to mitigate potential sources of bias, the evaluation employed the risk of bias criteria, considering the experimental nature of the studies, which pertained to randomized clinical trials.

Studies that demonstrated comprehensive data across all these domains were categorized as exhibiting a commendable level of methodological accuracy. Those displaying the presence of two to three factors were acknowledged as maintaining a reasonable standard of quality. Conversely, studies that lacked substantial data concerning the majority of factors, whether none or only one, were classified as manifesting a lower quality level as shown in Table 3 and Table 4.

**Table 3:** Assessment of risk of bias (low: < 6, medium: 6 to 12, high: > 12)

Domain	Lin et al. (2017) [13]	Poorni et al. (2017) [14]	Nagy et al. (2014) [15]	Huang et al. (2013) [16]	Narang et al. (2015) [17]	Jadhav et al. (2012) [18]	Alagl et al. (2017) [19]
Random sequence generation	1	1	1	3	2	1	1
Allocation concealment	1	1	2	2	2	2	2
Blinding of participants and personnel	1	1	2	2	2	2	3
Blinding of outcome assessment	1	1	1	2	1	2	2
Incomplete outcome data	1	2	1	3	1	1	1
Selective reporting	1	1	1	1	1	1	1
Other bias	1	1	1	1	1	1	1
Total	7	9	9	14	10	10	11

**Table 4:** Assessment of risk of bias (low: < 6, medium: 6 to 12, high: > 12)

Domain	Sharma et al. (2016) [20]	Shivashankar et al. (2017) [21]	Mittal et al. (2021) [22]	Ragab et al. (2019) [23]	Ulusoy et al. (2019) [24]	ElSheshtawy et al. (2020) [25]	Rizk et al. (2019) [26]	Ramachandran et al. (2020) [27]
Random sequence generation	1	1	1	1	1	1	1	1
Allocation concealment	1	1	2	1	2	1	1	1
Blinding of participants and personnel	2	1	1	3	3	3	3	2
Blinding of outcome assessment	2	1	1	1	1	1	1	1
Incomplete outcome data	1	1	1	1	1	1	1	1
Selective reporting	1	1	1	1	1	1	1	1
Other bias	1	1	1	1	1	1	1	1
Total	9	7	8	9	10	9	9	8

Results

Search and Selection

The article selection for this systematic review was conducted based on the PRISMA checklist. The literature search yielded 850 articles where 295 were obtained from other sources. A total of 555 duplicate articles were removed, and after adequate screening, 487 articles were retained. After the exclusion criteria were considered, full-text articles, which were 286 in number, were considered. Finally, 15 articles were elaborated in the present systematic review.

The age spectrum of individuals encompassed in the clinical trials ranged from seven to 20 years. In the majority of the conducted studies [13,14], sodium hypochlorite was the primary cleaning agent, with concentrations ranging from 1.5% to 3%. This solution was used for irrigation in both the apexification and revascularization groups. However, a specific study [15] deviated from this practice and utilized a combination of 3% hydrogen peroxide and 0.9% normal saline for cleaning purposes.

The revascularization therapy group consistently incorporated antibiotics, primarily utilizing a triple antibiotic paste, comprising ciprofloxacin, metronidazole, and one of the following: minocycline, clindamycin hydrochloride, or doxycycline. In some cases, only metronidazole was used to target specific bacterial concerns [15]. Additionally, a study conducted a comparison between the effectiveness of triple antibiotic paste and double antibiotic paste as intracanal medicaments [16].

Within the regenerative therapy group, blood clot scaffolds were predominant, often accompanied by injectable scaffolds. Mineral trioxide aggregate (MTA) was the most common scaffold sealant, featured in two studies, along with glass ionomer cement (GIC) used in an equal number of studies. One study used calcium hydroxide (CH) as the sealing material. Follow-up periods ranged from 12 to 18 months [16].

The clinical protocol involved removing the necrotic pulp, irrigating with sodium hypochlorite (NaOCl) or ethylenediamine tetraacetic acid (EDTA), limited dentin wall manipulation, applying double or triple antibiotic paste and calcium hydroxide over several weeks, introducing platelet concentrates into the root canal, solidifying the material, and sealing with biomaterial like mineral trioxide aggregate, glass ionomer cement, or bonded resin [17,18].

The radiographic assessment encompassed both qualitative and quantitative dimensions, employing techniques such as ConeBeam CT and periapical digital radiography. Measurements included periapical radiolucency, lesion size, overall radiographic root area, and occasionally root canal length [19].

The primary adverse outcomes identified in these studies encompassed root canal calcification and tooth discolouration. In a specific study [20], instances of partial blockage in pulp canals were detected in both the groups undergoing treatment and the control groups. Tooth discolouration was recorded in association with revascularization procedures in three studies [19-21], with one study highlighting a greater incidence of crown discolouration in the blood clot group compared to the PRP group. Apart from these effects, no further unfavourable events were reported in cases where immature necrotic teeth were treated using PRP, PRF, or blood clots.

Quality of Studies

Among the 16 included studies, 15 articles had a medium risk of bias and one article had a high risk of bias. The major methodologic limitations were unclear baseline characteristics, reliability, and time intervals. Two studies had a higher risk of bias with random sequence generation and in four articles, blinding of participants was not performed.

Discussion

In the field of endodontics, addressing the management of treatment for immature necrotic teeth characterized by incomplete root formation presents a complex and intricate challenge. These teeth often exhibit a substantial apical diameter and a disproportioned crown-to-root ratio, rendering conventional endodontic techniques for pulp treatment and subsequent restoration difficult and yielding suboptimal retention outcomes [22].

Regenerative endodontic therapy, an emerging approach known for promoting ongoing root development, has been the focus of several clinical trials [23,24]. However, contrasting research has indicated that regenerative endodontic therapy might not surpass alternative apexification techniques, with instances of increased adverse events and diminished success rates associated with regenerative endodontic therapy [25]. This lack of systematic analysis hinders the effective dissemination of innovative technologies and the informed selection of treatment approaches. Given the lack of consensus between apexification and regenerative endodontic therapy, this study aims to conduct an exhaustive systematic review to offer a scientifically informed reference for treatment decision-making.

When young permanent teeth with broad apical foramina experience pulp necrosis, the potential for infection to spread to periapical areas escalates, resulting in a complex healing environment. Effectively addressing periapical inflammation significantly influences treatment results. In the context of managing periapical periodontitis, two studies encompassing four groups yielded qualitative observations [26]. The success rates for treating periapical periodontitis in revascularization endodontic therapy groups varied from 60% to 100%, whereas both studies reported 100% success rates in the apexification groups. Potential factors contributing to this difference include lingering bacteria and instability of the scaffold [27].

In revascularization endodontic therapy, inducing apical bleeding is a widely employed method to introduce essential components for tissue regeneration. This procedure allows the inclusion of periodontal ligament stem cells, bone marrow mesenchymal stem cells, and Hertwig epithelial root sheath cells into the root canal space. Additionally, PRP and PRF are commonly incorporated into revascularization techniques [12]. PRP and PRF are rich in growth factors and platelets, which promote tissue healing and regeneration [14]. They can be integrated with the revascularization process to enhance the potential for successful root maturation. Furthermore, there is a mention of a more recent approach called tPRF or titanium-prepared PRF, which is a variation of PRF utilizing titanium tubes. tPRF is thought to provide a sustained release of growth factors and may further enhance the regenerative capacity of revascularization treatments [13].

## Conclusions

Drawing from our comprehensive systematic review, a comprehensive deduction can be drawn, indicating that both treatment protocols exhibit efficacy in addressing the recuperation of periapical periodontitis as well as the resolution of open apices. In terms of promoting root elongation and thickening, revascularization endodontic therapy showcases superior efficacy without engendering a greater likelihood of treatment invalidation. This finding implies that revascularization endodontic therapy holds potential as the preferred primary option in treating immature necrotic teeth characterized by substantial root maldevelopment. Nonetheless, for individuals harbouring elevated aesthetic expectations, the implementation of revascularization endodontic therapy necessitates prudent consideration, taking into account the patient's preferences as part of the treatment approach.
